# Comparison of 9.0 and 9.2 mm Flap Diameter Options of Femtosecond Laser In-Situ Keratomileusis for Hypermetropia and Hypermetropic Astigmatism

**DOI:** 10.1155/2019/5907645

**Published:** 2019-11-06

**Authors:** Kemal Ozulken, Cagri Ilhan

**Affiliations:** ^1^Department of Ophthalmology TOBB University, Ankara, Turkey; ^2^Hatay State Hospital, Antakya, Hatay, Turkey

## Abstract

**Aim:**

To compare the postoperative one-year outcomes of asphericity (Q) and high order aberration (HOA) values of 9.0 and 9.2 mm diameter flap groups in hypermetropia and hypermetropic astigmatism subjects who underwent femtosecond laser in-situ keratomileusis (LASIK).

**Materials and Methods:**

The study included 68 eyes of 34 patients. A femtosecond laser platform (Allegrato Wave, Wavelight AG, Erlangen, Germany) was used for flap cutting. Corneal stroma was ablated using Wavelight EX500 with wavefront-optimized profile (WaveLight GmbH, Erlangen, Germany). 9.0 mm flap diameter was randomly chosen for one eye, and 9.2 mm flap diameter was chosen for the fellow eye. Two eyes of the patients who used two different flap diameters were enrolled into two different groups. Corneal stroma was ablated using Wavelight EX500 with wavefront-optimized profile (WaveLight GmbH, Erlangen, Germany). Postoperative one-year outcomes of Q and HOA values of 9.0 and 9.2 mm diameter flap groups were compared statistically.

**Results:**

The preoperative manifest refraction spherical equivalents of the 9.0 and 9.2 mm diameter flap groups were 1.86 ± 1.81 D and 1.69 ± 1.99 D (*p*=0.754). No intraoperative or postoperative complications were observed. At postoperative one-year, Q values were 0.98 ± 0.13 D and 0.91 ± 0.15 D (*p*=0.029). HOAs including horizontal and vertical coma, horizontal and vertical trefoil, spherical aberration, and second order vertical coma were not significantly different (*p* > 0.05 for all). Total HOA values were 1.62 ± 0.14 and 1.40 ± 0.16, in the 9.0 and 9.2 mm diameter flap groups, respectively (*p* < 0.001).

**Conclusion:**

Both the 9.0 and 9.2 mm diameter flap options in femtosecond LASIK are equally safe and effective. Many of the HOA values are similar in both options, and better results were provided in terms of total HOA and Q values with the 9.2 mm diameter flap option. This study was registered with trial registration number 118-011.

## 1. Introduction

Laser refractive surgery is commonly used for correction of refractive errors including myopia, hypermetropia, astigmatism, and presbyopia [1]. New laser platforms have increased the accuracy and safety of the procedures with technological improvements and innovations [2]. Laser-assisted in-situ keratomileusis (LASIK) is a safe, effective, and predictable technique in correcting refractive errors [1]. With LASIK surgery, the corneal flap is created to ablate the stroma, and the central cornea is flattened to decrease the optical power in myopic subjects, while the central cornea is steepened to increase the optical power in hypermetropic subjects [3]. Femtosecond laser technology adopted this procedure as it offers an alternative way to create a corneal flap, and many studies have shown the superiority of femtosecond laser over microkeratome in terms of corneal recovery and visual outcomes [2–4]. Nowadays, femtosecond LASIK is a widely performed procedure all over the world [3]. The creation of the epithelial-stromal flap is one of the most crucial steps in LASIK surgery, and femtosecond laser provides corneal flap configuration by changing the morphology, depth, and diameter, thereby refining the postoperative results, which have been studied in recent years [5–8].

To the best of our knowledge, there is only one study [8] which investigated the flap diameters in patients with myopia, and there are no studies evaluating the flap diameters in hypermetropic patients. The aim of the study was to compare the postoperative one-year Q and high order aberration (HOA) values of 9.0 and 9.2 mm diameter flap groups in hypermetropia and hypermetropic astigmatism subjects who underwent femtosecond LASIK.

## 2. Materials and Methods

### 2.1. Design

This nonrandomized, comparative study was conducted between January 2016 and June 2018, in the refractive surgery department of an eye hospital. The study followed the tenets of the Declaration of Helsinki and was approved by the Local Ethics Committee of TOBB ETU Medical School (KAEK 118-011). Informed consent was obtained from each patient before surgery after detailed explanation of the surgical procedures.

### 2.2. Subjects

The study included 68 eyes of 34 patients who underwent femtosecond LASIK. All subjects met the following inclusion criteria: ages between 18 and 46 years, positive spherical refractive error <6 diopter (D) and cylindrical refractive error <4 D, stable refractive error for the previous year, and best corrected visual acuity (BCVA) ≥0.00 log MAR. Exclusion criteria were defined as a history of ocular surgery or trauma, anisometropia, irregular astigmatism on corneal topography, residual stromal thickness of <300 *μ*m at the thinnest point, >6.0 mm diameter scotopic pupil size, pregnancy or lactation, or systemic abnormalities such as diabetes mellitus, collagen vascular diseases, or autoimmune diseases.

### 2.3. Clinical Evaluations

The subjects underwent detailed preoperative ophthalmological examinations after contact lens discontinuation for at least 2 weeks. Manifest and objective refraction were determined and uncorrected, and the BCVA were determined using a Snellen chart, and decimal values were converted to log MAR for statistical analysis. Slit-lamp biomicroscopy and dilated fundus examinations were performed, and intraocular pressure (IOP) was measured with a pneumotonometer.

Aberration measurements and corneal topography were performed using the WaveLight®Oculyzer II (Pentacam, Germany). The asphericity calculation was made using the Placido-based Allegrato Topolyzer (version 1.59, Alcon Laboratories, Inc). Total corneal HOAs including horizontal and vertical coma (Z(3, 1), Z(3, −1)), horizontal and vertical trefoil (Z(3, 3), Z(3, −3)), spherical aberration (Z(4, 0)), second order vertical coma (Z(5, −1)), and total HOA in the Zernike analysis were analyzed. The Topolyzer system performs the Zernike analyzes with measured height data. For each Zernike polynomial, the system calculates a coefficient which describes the contribution of that polynomial to the height data. Total corneal aberrations, calculated from the elevation values by the Pentacam software, were evaluated in the 6.0 mm diameter central area with respect to the pupil center in a dark environment, and the pupil was not dilated. These measurements were taken preoperatively and again at the end of the first year postoperatively.

### 2.4. Surgical Procedures

All surgeries were performed by a single experienced refractive surgeon (KO) at one center. In the operation room, topical proparacaine hydrochloride 0.5% (Alcaine, Alcon, Fort Worth, TX, USA) was instilled for topical anesthesia, and the right eye was operated on first. The eyelids were opened using a wire lid speculum, and the standard preoperative asepsis protocol was applied. The Allegrato Wave laser platform (Wavelight AG, Erlangen, Germany) was used to create a flap thickness of 120 *μ*m with a 70° angled side cut. One of the 9.0 mm and 9.2 mm flap diameters were determined randomly for one eye, and the other diameter was then applied to the fellow eye of each subject. The optical zone diameter was 7.0 mm, transition zone diameter was 0.95 mm, and total ablation zone diameter was 8.9 mm for all eyes. Bed spot and bed line laser separations were 8 *μ*m, and side spot and side line laser separations were 5 and 3 *μ*m, respectively. After drying the stromal bed, excimer laser ablation was performed using a Wavelight EX500 with wavefront-optimized ablation profile (WaveLight GmbH, Erlangen, Germany) and the Topolyzer Vario (Wavelight GmbH, Erlangen, Germany). The bed was thoroughly irrigated with saline, and the flap was repositioned on the stromal bed. The patients were blinded as to which flap diameter was determined for each eye.

As topical postoperative medication, moxifloxacin 0.5% (Vigamox, Alcon, Fort Worth, TX) 3 times a day for 1 week and dexamethasone (Maxidex, Alcon, Fort Worth, TX) at decreasing dosage starting from 5 times a day for 3 weeks were prescribed. Preservative-free artificial tear drops (Refresh, Allergan, Irvine, CA) were added 8 times a day for 2 months. All the patients were instructed not to rub their eyes or go swimming for the first month to prevent flap displacement or infectious keratitis.

### 2.5. Statistical Analysis

The data obtained from the study were analyzed using the Statistical Package for the Social Sciences (SPSS) 24.0 software (IBM Corp., New York, USA). Descriptive statistics were presented as mean ± standard deviation (SD). The normal distribution of the variables was tested using the Kolmogorov–Smirnov test. The nonparametric tests were used in analysis as the numerical data did not conform to normal distribution. The preoperative and postoperative variables of the same eye were compared using the Wilcoxon test. Statistical significance was set at *p* < 0.05 for all tests.

## 3. Results

**Figure 1:**
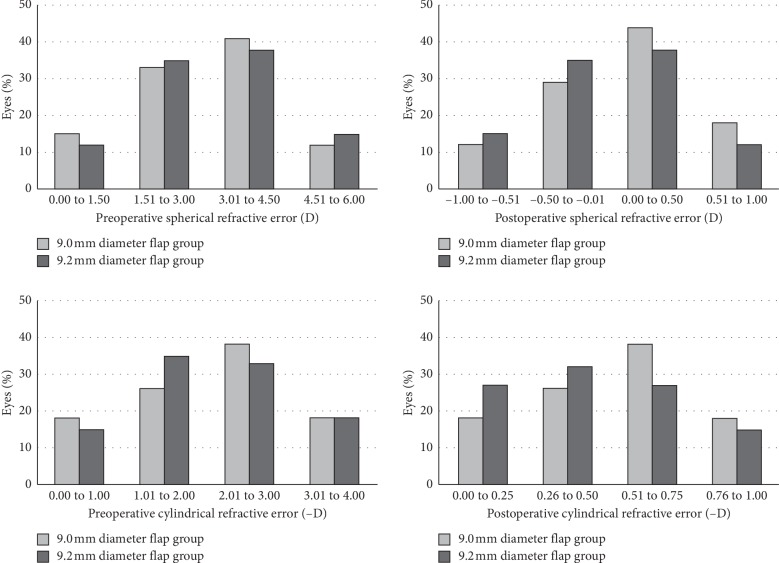
The demonstration of preoperative and postoperative refractive outcomes of the groups.

The mean age of the patients (19 female and 15 male) was 26.82 ± 6.21 (18–46 years). The preoperative spherical refractive errors were 3.26 ± 1.75 D and 2.67 ± 1.48 D, and the preoperative cylindrical refractive errors were 2.82 ± 1.34 D and 1.96 ± 1.60 D, in the 9.0 and 9.2 mm diameter flap groups, respectively (*p* > 0.05 for both) (Figure 1). The other preoperative clinical findings including manifest refraction spherical equivalent, BCVA, flat and steep keratometries, IOP, central corneal thickness, and residual stromal thickness were not significantly different (*p* > 0.05 for all). In the intraoperative period, the vacuum was not released during flap creation, and all surgeries were performed successfully. No intraoperative or postoperative complications developed in any case, including flap hinge, bleeding in the corneal limbus, or flap decentration. In both the 9.0 and 9.2 mm diameter flap groups, a significant improvement was determined in the clinical findings at one year after surgery (*p* < 0.001 for all). The postoperative one-year clinical findings were not significantly different in the 9.0 and 9.2 mm diameter flap groups (*p* > 0.05 for all). The clinical characteristics of the groups are shown in Table 1.

**Figure 2:**
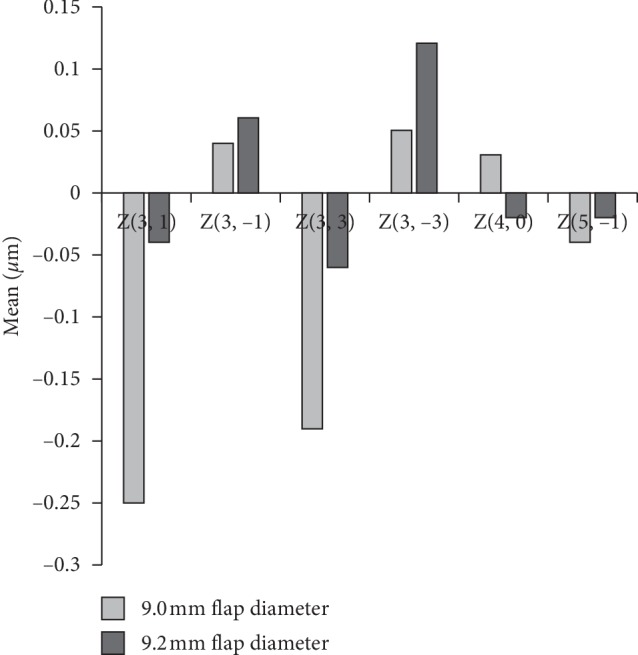
The similarity of the high order aberrations of the 9.0 and 9.2 mm diameter flap groups. *Z*(3, 1): horizontal coma; *Z*(3, −1): vertical coma; *Z*(3, 3): horizontal trefoil; *Z*(3,−3): vertical trefoil; *Z*(4, 0): spherical aberration; and *Z*(5, −1): second order vertical coma.

The postoperative one-year results of Q values were 0.98 ± 0.13 D and 0.91 ± 0.15 D in the 9.0 and 9.2 mm diameter flap groups, respectively, and the difference was significant (*p*=0.029). The postoperative one-year HOAs including horizontal and vertical coma, horizontal and vertical trefoil, spherical aberration, and second order vertical coma were not significantly different in the two groups (*p* > 0.05 for all) (Figure 2). The postoperative one-year total HOA was 1.62 ± 0.14 and 1.40 ± 0.16 in the 9.0 and 9.2 mm diameter flap groups, respectively, and this difference was found to be significant (*p* < 0.001) (Table 2).

## 4. Discussion

In the LASIK procedure, the corneal flap is the most important determinant of a successful outcome [3–6]. Femtosecond laser technology has decreased the time spent in cutting the flap, which can be made more accurately and at a more predictable depth than those created by mechanical microkeratomes, and thus has improved the quality of the stromal bed [4, 9]. In addition, a customized corneal flap configuration can be provided by femtosecond laser technology in many directions [3–5,9]. Flap thickness is one of the most important parameters, and a previous study has shown that many complications including corneal haze, flap tear, bubble escape, free flap, flap fold, diffuse lamellar keratitis, and epithelial ingrowth occur more commonly in thin flaps [10]. However, no differences were found between thin and thick flaps in terms of contrast sensitivity and total HOA [7, 11]. In the current study, a comparison was made of different flap diameters which can be set automatically by the femtosecond laser device. When a small flap diameter is used, it is necessary to take additional precautions to avoid damage to the hinge because the hinge is close to the ablation area [9, 12]. However, if a wide flap diameter is set, additional protective measures are not required because the corneal hinge will be further away from the ablation area, and this results in a shorter duration of surgery [8, 12]. Moreover, because the corneal hinge is far from the center of ablation, the quality of ablation is better as the corneal stroma remains dry during the laser shots [8, 12]. With larger diameter flaps, more space is created for myopic, astigmatic, and especially hypermetropic ablation so that a larger optical zone and a blend zone can be adjusted [3, 12]. Re-epithelialization is also faster with the use of larger flaps since epithelial cells are produced from peripheral cornea [9, 13]. The disadvantages of larger flaps are the risk of bleeding because they are closer to limbal vessels [12]. In addition, previous studies have shown that the lamellar and fibrillar distribution of collagen in the peripheral corneal stroma has more cohesive tensile strength [10, 12, 13]. In contrast, there are studies indicating that smaller corneal flap has better corneal stability because of less damage to the peripheral cornea [8]. We think that further studies are needed to support the theory that smaller flap diameter provides better corneal stability. In the current study, we investigated whether there is any difference between 9.0 and 9.2 mm diameter flap groups in terms of the postoperative one-year clinical findings, asphericity, and HOAs. The study results demonstrated that both 9.0 and 9.2 mm diameter flaps are similarly safe, and no significant intraoperative or postoperative complications were observed in any subject. Both the 9.0 and 9.2 mm flap diameters were also seen to be similarly effective when considering the postoperative one-year clinical findings, including spherical and cylindrical refractive errors, manifest refraction spherical equivalent, BCVA, flat and steep keratometry values, IOP, and central corneal thickness.

In the current study results, the HOAs including trefoil, coma, and spherical aberration were not statistically different in the 9.0 and 9.2 mm diameter flap groups. The Topolyzer system calculates a total HOA from the Zernike coefficients. Values exceeding 1.0 indicate that there are atypical wave components. In the current study results, the total HOA was different in the two groups, and it was closer to 1.0 in the 9.2 mm diameter flap group. Although there was no statistically significant difference in the two groups with different flap sizes in terms of Zernike polynomials, the difference in total HOAs showed that postoperative HOAs were not completely the same. This is relatively new information because Zhang et al. [8] reported that Zernike polynomials are not statistically different in small and big flap groups, but they used another aberrometer device which does not calculate the total HOA in subjects who underwent femtosecond LASIK in which the corneal flap diameters were set as 8.1 and 8.6 mm. In current study, lower asphericity value in eyes with 9.2 mm flap diameter is another finding supporting the superiority of 9.2 mm diameter flap. The better results in asphericity and total HOA, provided by the 9.2 mm diameter flap in this study, indicate that better visual quality can be achieved with the 9.2 mm flap diameter option.

The excimer laser systems basically provide customized reshaping of cornea according to the refractive error of the subjects [1, 2]. Central corneal ablation is performed in myopic refractive error, and central flattening is achieved [14]. In contrast, peripheral ablation is performed in hypermetropic subjects, and central steepening is achieved [14]. In this regard, it may be thought that the ideal subjects should have hypermetropia or hypermetropic astigmatism to be able to investigate the differences in corneal topographic parameters after LASIK surgery with different flap diameters because ablation is more intense when performed in the peripheral cornea, and the edges of the surgical area become more important in these subjects. There is only one study in literature that has compared postoperative visual quality based on different sizes of corneal flaps, and this study only included subjects with myopic refractive error [8]. Therefore, this study can be considered of value as the first to discuss as a relatively new topic.

The similarity in many Zernike polynomials in the 9.0 and 9.2 mm diameter flap groups could be related to methodological restriction. The Topolyzer system evaluates in the 6.0-mm-diameter central area of the cornea and does not measure the areas between 6.0 and 9.0 mm or 6.0 and 9.2 mm. Therefore, the system provides limited information about the corneal topographic evaluation, and this method can be considered an important limitation of the study. In addition, there was no investigation of the clinical importance of the statistically significant different results, and therefore it is not known whether the visual quality of the eyes with a 9.0 mm flap had a negative effect on daily tasks. Further researches will be able to provide more reliable results if differences in corneal topographic parameters are clarified using another ideal system measuring all the corneal surface. In addition, studies evaluating visual quality parameters such as glare, halo, night vision, or contrast sensitivity would be useful to determine the clinical significance of the results of this study.

In conclusion, both the 9.0 and 9.2 mm diameter flap options in femtosecond LASIK seems to be safe and effective based on clinical findings. Although many of the Zernike polynomials are similar in both options, better results can be provided in terms of total HOA and Q values with the 9.2 mm diameter flap option.

## Figures and Tables

**Table 1:** The comparison of demographic and clinical characteristics of the 9.0 (*n* = 34) and 9.2 (*n* = 34) mm diameter flap groups.

	9.0 mm diameter flap group (mean ± SD)			9.2 mm diameter flap group (mean ± SD)		
	Preoperative value	Postoperative value	*p* value	Preoperative value	Postoperative value	*p* value	*p* value
Spherical RE (D)	3.26 ± 1.75 (0.75 to 5.75)	0.17 ± 0.56 (−0.75 to 1.00)	<0.001	2.67 ± 1.48 (0.75 to 5.75)	0.01 ± 0.35 (−0.50 to 0.75)	<0.001	^†^0.177 ^‡^0.274
Cylindrical RE (D)	−2.82 ± 1.34 (−3.75 to 0.00)	−0.55 ± 0.23 (−1.00 to −0.25)	<0.001	−1.96 ± 1.60 (−3.75 to 0.00)	−0.35 ± 0.20 (−0.75 to 0.00)	<0.001	^†^0.059 ^‡^0.055
MRSE (D)	1.86 ± 1.81 (0.75 to 5.50)	−0.10 ± 0.53 (−1.00 to 0.63)	<0.001	1.69 ± 1.99 (0.75 to 5.00)	−0.17 ± 0.33 (−0.75 to 0.63)	<0.001	^†^0.754 ^‡^0.533
BCVA (log MAR)	−0.13 ± 0.12 (0.00 to −0.30)	−0.07 ± 0.09 (0.00 to −0.30)	<0.001	−0.10 ± 0.06 (0.00 to −0.30)	−0.05 ± 0.14 (0.00 to −0.30)	<0.001	^†^0.306 ^‡^0.422
K1 flat (D)	41.20 ± 1.84 (37.01 to 44.29)	43.47 ± 1.63 (41.10 to 47.10)	<0.001	41.08 ± 1.93 (37.29 to 45.30)	42.98 ± 2.12 (40.20 to 47.80)	<0.001	^†^0.915 ^‡^0.114
K2 steep (D)	44.32 ± 1.68 (40.61 to 47.87)	44.50 ± 1.68 (41.50 to 48.10)	<0.001	43.37 ± 1.99 38.18 to 46.19)	43.63 ± 1.99 (40.90 to 48.40)	<0.001	^†^0.054 ^‡^0.052
IOP (mmHg)	14.79 ± 4.58 (10 to 21)	13.67 ± 3.27 (10 to 21)	<0.001	15.88 ± 2.45 (10 to 21)	12.85 ± 2.20 (10 to 21)	<0.001	^†^0.080 ^‡^0.221
CCT (*μ*m)	549.30 ± 32.40 (496 to 614)	502.00 ± 44.57 (422 to 599)	<0.001	548.76 ± 31.98 (503 to 628)	522.15 ± 45.30 (405 to 616)	<0.001	^†^0.965 ^‡^0.070
Residual stroma (*μ*m)	359.33 ± 46.69 (302 to 446)			369.53 ± 30.84 (321 to 430)			0.147

SD: standard deviation; RE: refractive error; D: diopter; MRSE: manifest refraction spherical equivalent; BCVA: best corrected visual acuity; K: keratometry; IOP: intraocular pressure; CCT: central corneal thickness; ^†^the comparison of the preoperative values; ^‡^the comparison of the postoperative values.

**Table 2:** The comparison of the postoperative one-year Q value and HOAs of the 9.0 (*n* = 34) and 9.2 (*n* = 34) mm diameter flap groups.

	9.0 mm diameter flap group (mean ± SD)	9.2 mm diameter flap group (mean ± SD)	*p* value
Q value (D)	0.98 ± 0.13 (0.65 to 1.23)	0.91 ± 0.15 (0.61 to 1.21)	0.029
*Z*(3, 1) (*μ*m)	−0.25 ± 0.47 (−1.25 to 0.57)	−0.04 ± 0.39 (−0.79 to 0.86)	0.186
*Z*(3, −1) (*μ*m)	0.04 ± 0.28 (−0.37 to 0.51)	0.06 ± 0.36 (−0.99 to 0.56)	0.603
*Z*(3, 3) (*μ*m)	−0.19 ± 0.28 (−0.69 to 0.43)	−0.06 ± 0.30 (−0.85 to 0.58)	0.056
*Z*(3, −3) (*μ*m)	0.05 ± 0.25 (−0.45 to 0.38)	0.12 ± 0.34 (−0.73 to 0.96)	0.448
*Z*(4, 0) (*μ*m)	0.03 ± 0.36 (−0.87 to 0.51)	−0.02 ± 0.52 (−0.99 to 0.85)	0.866
*Z*(5, −1) (*μ*m)	−0.04 ± 0.17 (−0.33 to 25)	−0.02 ± 0.17 (−0.31 to 0.25)	0.535
Total HOA	1.62 ± 0.14 (1.30 to 1.80)	1.40 ± 0.16 (1.10 to 1.60)	<0.001

HOA: high order aberration; SD: standard deviation; D: diopter.

## Data Availability

The data used to support the findings of this study are available from the corresponding author upon request.
